# A New Privacy-Preserving Handover Authentication Scheme for Wireless Networks

**DOI:** 10.3390/s17061446

**Published:** 2017-06-20

**Authors:** Changji Wang, Yuan Yuan, Jiayuan Wu

**Affiliations:** 1 School of Information Science and Technology, Guangdong University of Foreign Studies, Guangzhou 510420, China; 2 School of Finance, Guangdong University of Foreign Studies, Guangzhou 510420, China; 200711647@oamail.gdufs.edu.cn; 3 School of Data Science and Computer, Sun Yat-sen University, Guangzhou 510420, China; wujy26@mail2.sysu.edu.cn

**Keywords:** wireless networks, handover authentication, identity-based signature, blind signature, authenticated key establishment, elliptic curve cryptography

## Abstract

Handover authentication is a critical issue in wireless networks, which is being used to ensure mobile nodes wander over multiple access points securely and seamlessly. A variety of handover authentication schemes for wireless networks have been proposed in the literature. Unfortunately, existing handover authentication schemes are vulnerable to a few security attacks, or incur high communication and computation costs. Recently, He et al. proposed a handover authentication scheme PairHand and claimed it can resist various attacks without rigorous security proofs. In this paper, we show that PairHand does not meet forward secrecy and strong anonymity. More seriously, it is vulnerable to key compromise attack, where an adversary can recover the private key of any mobile node. Then, we propose a new efficient and provably secure handover authentication scheme for wireless networks based on elliptic curve cryptography. Compared with existing schemes, our proposed scheme can resist key compromise attack, and achieves forward secrecy and strong anonymity. Moreover, it is more efficient in terms of computation and communication.

## 1. Introduction

With the rapid development of the wireless internet access techniques, more and more mobile services have appeared, which provide a more convenient life to people. For instance, wireless local area networks (WLANs) offer convenient access to network services [1], vehicular ad hoc networks (VANETs) provide great opportunity for collaborative traffic information exchange [2], wireless sensor networks (WSNs) can monitor physical or environmental information in real time [3]. Handover authentication is essential to overcome the geographical coverage limit of each access point, which enables mobile nodes (e.g., Laptop, PDA, smart phone and vehicle) to securely and seamlessly roam over multiple access points [4].

**Figure 1:**
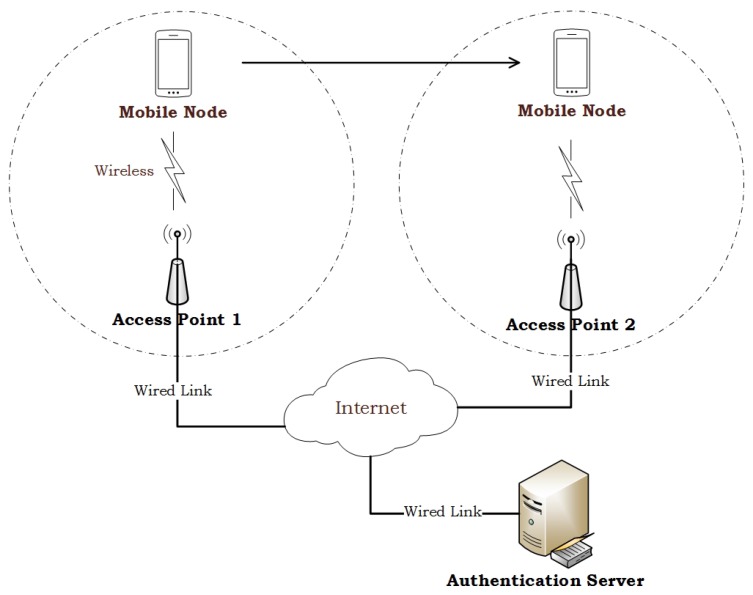
Handover authentication scenario.

Generally, a handover authentication scheme involves three participants: mobile nodes (MNs), access points (APs) and the authentication server (AS). An MN registers to the AS, and then connects to any AP to access its subscription services. An AP acts a guarantor for vouching for an MN as a legitimate subscriber. When an MN moves from the current AP (e.g., ${\mathrm{AP}}_{1}$) into a new AP (e.g., ${\mathrm{AP}}_{2}$), it will trigger the execution of handover authentication at ${\mathrm{AP}}_{2}$. Then, ${\mathrm{AP}}_{2}$ verifies whether the MN is authorized user or not. If the MN is an unauthorized user, ${\mathrm{AP}}_{2}$ will reject the MN’s access request. If the MN is an authorized user, a session key will be established simultaneously for protecting data traffic between the MN and AP2. Figure 1 illustrates a typical handover authentication scenario.

Efficiency and security are two major challenges faced by researchers to design handover authentication scheme in wireless networks. On the one hand, the handover authentication process should be fast enough to cope with time limitation of handover, but MNs are generally constrained in terms of energy supply, bandwidth and processing capability. Therefore, a handover authentication scheme for wireless networks should be efficient in terms of communication and computation. On the other hand, security and privacy have become increasingly important in mobile computing, particularly in the context of handover authentication schemes as they relate to the MN’s credential information.

As a promising seamless access control technology, handover authentication schemes have received much attention in recent years [4,5,6,7,8,9,10,11]. He et al. [4] proposed a smart-card based handover authentication scheme, which requires ${\mathrm{AP}}_{2}$ to contact AS who vouches for the MN’s legitimacy, and there are four messages exchanged between an MN, ${\mathrm{AP}}_{1}$ and ${\mathrm{AP}}_{2}$ when an MN moves from ${\mathrm{AP}}_{1}$ into ${\mathrm{AP}}_{2}$. Obviously, this will result in more computation and communication delay, especially if the AS is often located in a remote location. Later, He et al. [5] proposed a privacy-preserving handover authentication scheme that ${\mathrm{AP}}_{2}$ does not communicate with the AS, but there are still three message exchanges between the MN and ${\mathrm{AP}}_{2}$ for mutual authentication and key establishment. To improve the communication efficiency and reducing the burden on the AS, He et al. [6] proposed a secure handover authentication scheme named PairHand. Instead of relying on the participation of the AS, PairHand only requires two handshakes between the MN and ${\mathrm{AP}}_{2}$ for mutual authentication and key establishment. Furthermore, PairHand uses a pool of shorter-lived pseudonyms to protect users’ privacy. Unfortunately, they soon found that PairHand is vulnerable to private key compromise attack [7], where an adversary can recover any MN’s private key. He et al. [7] then proposed an improved PairHand by replacing the prime *q* order bilinear group with a composite *n* order bilinear group. However, Yeo et al. [8] showed that He et al.’s improved PairHand is still vulnerable to private key compromise attack, even worse, an adversary is able to compute the master key when prime factors of *n* are all relatively small. However, they did not give any effective solutions to resist a private key compromise attack. Subsequently, Tsai et al. [9] and Wang et al. [10] presented two handover authentication schemes from prime-order bilinear pairings to resist the private key compromise attack, respectively. However, both Tsai et al.’s scheme [9] and Wang et al.’s scheme [10] can not achieve forward secrecy and are vulnerable to known session key attacks. Recently, Li et al. [11] proposed a handover authentication scheme without bilinear pairings. However, Chaudhry et al. [12] found that Li et al.’s scheme cannot withstand access point impersonation attacks.

In this paper, we further analyze the security of the improved PairHand and show that the improved PairHand does not meet forward secrecy and strong anonymity. Next, we propose a new efficient handover authentication protocol without bilinear pairings that fixes the security flaws in PairHand. Our main approach is to integrate Pointcheval and Stern’s blind signature scheme [13], Chatterjee et al.’s identity-based signature scheme [14], and Yasmin et al.’s identity-based authenticated key establishment protocol [15] into a handover authentication scheme. Compared to existing handover authentication schemes, our proposed scheme is more efficient in terms of computation and communication, and achieves escrow-free, MN forward secrecy, MN anonymity and untraceability. There is only one-pass message exchange between the MN and AP for mutual authentication and key establishment. In particular, batch verification for handover authentication is also achieved, and no bilinear pairing computation is required in our proposed handover authentication scheme.

This paper is organized as follows. We introduce some necessary preliminary work in Section 2. Next, we review He et al.’s improved PairHand and show that the improved PairHand can not satisfy required security properties in Section 3. We describe our new handover authentication scheme in Section 4, and present security and efficiency analysis of our proposed scheme in Section 5. Finally, we conclude our work in Section 6.

## 2. Preliminaries

To facilitate further description, we introduce notations in Table 1.

### 2.1. Bilinear Pairings and Complexity Assumptions

Let $G_{1}$ be an additive cyclic group generated by *P*, with prime order *q*, and $G_{2}$ be a multiplicative group of the same order *q*. A bilinear pairing is a map $\hat{e}:G_{1}\times G_{1}\to G_{2}$ with the following properties:

- Bilinearity: For $a\overset{\$}{\leftarrow }Z_{q}$ and $b\overset{\$}{\leftarrow }Z_{q}$, we have $\hat{e}\left(\left[a\right]P,\left[b\right]P\right)=\hat{e}{\left(P,P\right)}^{ab}$.
- Non-degeneracy: $\hat{e}\left(P,P\right)\neq 1$, where 1 is the identity element of $G_{2}$.
- Computability: There is an efficient algorithm to compute $\hat{e}\left(P_{1},P_{2}\right)$ for $P_{1}\overset{\$}{\leftarrow }G_{1}$ and $P_{2}\overset{\$}{\leftarrow }G_{1}$.

Typically, $G_{1}$ will be a subgroup of the group of points on the elliptic curve over a finite field, $G_{2}$ will be a subgroup of the multiplicative group of a related finite field and the map $\hat{e}$ will be derived from the Weil or Tate pairing on the elliptic curve.

Let $E_{p}\left(a,b\right)$ be a set of elliptic curve points over the prime field $F_{p}$, defined by the non-singular elliptic curve equation $y^{2}=x^{3}+ax+b\;\mathrm{mod}\;p$, together with a special point at infinity O, where $a,b\in F_{p}$ and $4a^{3}+27b^{2}\;\mathrm{mod}\;p\neq 0$. This set together with the group operation of elliptic curve is an Abelian group, with the point at infinity as identity element.

Let $P\in E_{p}\left(a,b\right)$ be a point of prime order *q*, and $G_{1}$ be a subgroup generated by *P*, i.e., $G_{1}\overset{\mathrm{def}}{=}\langle P\rangle$.

**Definition** **1.** Given $Q\in G_{1}$, the elliptic curve discrete logarithm problem (ECDLP) for $G_{1}$ is to find the integer x, 1≤x≤q, such that Q=[x]P.

The advantage of an adversary A in breaking the ECDLP is defined by

$${\mathrm{Adv}}_{A}^{\mathrm{ECDLP}}\left(1^{\kappa }\right)=\mathrm{Pr}\left[A\left(P,Q=\left[x\right]P\right)=x|x\overset{\$}{\leftarrow }Z_{q}^{*}\right].$$

We say that the elliptic curve discrete logarithm assumption (ECDLA) holds for the group $G_{1}$ if, for any probabilistic polynomial-time adversary A, the advantage ${\mathrm{Adv}}_{A}^{\mathrm{ECDLP}}\left(1^{\kappa }\right)$ is a negligible function in the security parameter κ.

**Definition** **2.** Given $\left(P,\left[a\right]P,\left[b\right]P\right)\in G_{1}^{\left(3\right)},$ where $a,b\overset{\$}{\leftarrow }Z_{q}^{*}$, the elliptic curve computational Diffie–Hellman problem (ECCDHP) for the group $G_{1}$ is to compute [ab]P.

The advantage of an adversary A in breaking ECCDHP is defined by

$${\mathrm{Adv}}_{A}^{\mathrm{ECCDH}}\left(1^{\kappa }\right)=\mathrm{Pr}\left[A\left(P,\left[a\right]P,\left[b\right]P\right)=\left[ab\right]P|a,b\overset{\$}{\leftarrow }Z_{q}^{*}\right].$$

We say that the elliptic curve computational Diffie–Hellman assumption (ECCDHA) holds for $G_{1}$ if for any probabilistic polynomial-time adversary A, the advantage ${\mathrm{Adv}}_{A}^{\mathrm{ECCDH}}\left(1^{\kappa }\right)$ is a negligible function in the security parameter κ.

### 2.2. Pointcheval and Stern’s Blind Signature Scheme

Blind signatures allow a user to obtain signatures from a signer on any message, in such a way that the signer learns nothing about the message that is being signed, and no one can derive a link between one of the messages which the signer has received and a valid blind signature, except the signature requester. Pointcheval and Stern [13] proposed an efficient blind signature scheme based on Schnorr signature scheme, which proved to be secure in the random oracle model under the ECDLA. Pointcheval and Stern’s blind signature scheme is described as follows.

- **Setup**: A trusted authority generates an elliptic curve group $G_{1}$ of prime order *q* with a generator *P*, and publishes domain parameters $params=\langle P,q,G_{1},H_{1}\rangle$.
- **KeyGen**: The signer chooses $x\overset{\$}{\leftarrow }Z_{q}^{*}$, computes Y=[−x]P, sets the secret signing private key as *x* and the corresponding public verification key as *Y*.
- **Sign**: In order to get the signature of a message $m\in {\left\{0,1\right\}}^{*}$, a requester asks the signer to initiate a communication. The signer chooses $k\overset{\$}{\leftarrow }Z_{q}^{*}$, computes and sends the commitment R=[k]P to the requester. Upon receiving the commitment, the requester blinds it with two random elements $\alpha ,\beta \overset{\$}{\leftarrow }Z_{q}^{*}$ into $R^{\prime }=R+\left[\alpha \right]P+\left[\beta \right]Y$, computes $c^{\prime }=H_{1}\left(m,R^{\prime }\right)$ and sends the challenge $c=c^{\prime }-\beta \;\mathrm{mod}\;q$ to the signer. Then, the signer returns a values s=k+cxmodq to the requester. Finally, the requester verifies the following equation holds or not:
  $$\left[s\right]P+\left[c\right]Y\overset{?}{=}R.$$
  If it holds, then the requester computes $s^{\prime }=s+\alpha \;\mathrm{mod}\;q$, and obtains a blind signature $\left(c^{\prime },s^{\prime }\right)$ that is signed by the signer for the unknown message *m*.
- **Verify**: Anyone can verify that the pair $\left(c^{\prime },s^{\prime }\right)$ is a valid Schnorr signature of *m* since it satisfies $c^{\prime }=H_{1}\left(m,R^{\prime }\right)$, where $R^{\prime }=\left[s^{\prime }\right]P+\left[c^{\prime }\right]Y$.

Blind signature schemes have been widely used in systems that guarantee participants’ anonymity. We will use the above blind signature scheme in our handover authentication scheme to guarantee MNs’ strong anonymity.

### 2.3. Improved Galindo and Garcia’s Identity-Based Signature Scheme

Galindo and Garcia [16] proposed a lightweight identity-based signature scheme named GG-IBS in Africacrypt 2009. It is recognized as one of the most efficient identity-based signature schemes until now because no complicated bilinear pairings are required in the GG-IBS scheme. We describe the GG-IBS scheme as follows.

- **Setup**: A trusted authority named PKG first generates an elliptic curve group $G_{1}$ of prime order *q* with a generator *P*, chooses $s\overset{\$}{\leftarrow }Z_{q}^{*}$ and computes $P_{\mathrm{pub}}=\left[s\right]P$. Finally, the PKG sets the master secret key msk=s and publishes the master public key $mpk=\langle G_{1},q,P,P_{\mathrm{pub}},H_{1},H_{2}\rangle$.
- **Extract**: A user submits a private key request with his/her identity information $\mathrm{id}\in {\left\{0,1\right\}}^{*}$ to the PKG. Upon receiving the request, the PKG chooses $r_{\mathrm{id}}\overset{\$}{\leftarrow }Z_{q}^{*}$, computes $R_{\mathrm{id}}=\left[r_{\mathrm{id}}\right]P$, $c=H_{1}\left(\mathrm{id},R_{\mathrm{id}}\right)$ and $sk_{\mathrm{id}}=r_{\mathrm{id}}+cs\;\mathrm{mod}\;q$. Finally, the PKG sends $\left(sk_{\mathrm{id}},R_{\mathrm{id}}\right)$ to the user via a secure channel. Upon receiving the response message, the user computes $c=H_{1}\left(\mathrm{id},R_{\mathrm{id}}\right)$ and checks the following equation:
  $$\left[sk_{\mathrm{id}}\right]P\overset{?}{=}R_{\mathrm{id}}+\left[c\right]P_{\mathrm{pub}}.$$
  If it holds, the user keeps the tuple $\left(sk_{\mathrm{id}},R_{\mathrm{id}}\right)$ as his/her identity-based signing private key. The corresponding public key can be computed as $R_{\mathrm{id}}+H_{1}\left(\mathrm{id},R_{\mathrm{id}}\right)P_{\mathrm{pub}}$.
- **Sign**: To sign a message *m*, the signer with identity id and signing private key $sk_{\mathrm{id}}$ chooses $a\overset{\$}{\leftarrow }Z_{q}^{*}$, computes $c=H_{1}\left(\mathrm{id},R_{\mathrm{id}}\right)$, A=[a]P, $d=H_{2}\left(m,A,c\right)$ and $b=a+sk_{\mathrm{id}}d\;\mathrm{mod}\;q$. Finally, the signer sets $\sigma =(b,R_{\mathrm{id}},A)$ as his/her signature on *m*.
- **Verify**: Given the signer’s identity id, a pair of message *m* and signature $\sigma =(b,R_{\mathrm{id}},A)$, anyone can compute $c=H_{1}\left(\mathrm{id},R_{\mathrm{id}}\right)$ and $d=H_{2}\left(m,A,c\right)$, check the following equation:
  $$\left[b\right]P\overset{?}{=}A+\left[cd\right]P_{\mathrm{pub}}+\left[d\right]R_{\mathrm{id}}.$$
  If it holds, the verifier accepts the signature and outputs true. Otherwise, outputs ⊥.

Chatterje et al. [14] proved that the GG-IBS scheme is existentially unforgeable under adaptively chosen identity and message attacks (EUF-ID-CMA) in the random oracle model under the ECDLA. We will use the GG-IBS scheme in our handover authentication scheme to provide mutual authentication between the AP and MN.

### 2.4. Yasmin et al.’s Identity-Based One-Pass Authenticated Key Establishment Protocol

Yasmin et al. [15] proposed a pairing-free, one-pass authenticated key establishment protocol. There are three algorithms in Yasmin et al.’s protocol: Setup, Extract and Key Exchange. The Setup algorithm and Extract algorithm are the same as those in the GG-IBS scheme. Here, we only describe the Key Exchange algorithm as follows.

- Alice, the initiator of the protocol, chooses $\ell \overset{\$}{\leftarrow }Z_{q}^{*}$, computes $L=[\ell sk_{{\mathrm{id}}_{A}}]P$, $c_{B}=H_{1}\left({\mathrm{id}}_{B},R_{{\mathrm{id}}_{B}}\right)$ and $PK_{{\mathrm{id}}_{B}}=c_{B}P_{\mathrm{pub}}+R_{{\mathrm{id}}_{B}}$, sets the shared session key $K_{A,B}=\mathrm{KDF}\left(\left[\ell sk_{{\mathrm{id}}_{A}}\right]PK_{{\mathrm{id}}_{B}}\right)$. Then, Alice deletes *L* and *ℓ*. Finally, Alice sends $\left(L,{\mathrm{id}}_{A},{\mathrm{id}}_{B},\sigma \right)$ to the receiver Bob, where σ is Alice’s identity-based signature on the ephemeral public key *L* together with Alice’s identity ${\mathrm{id}}_{A}$ and Bob’s identity ${\mathrm{id}}_{B}$.
- Bob verifies the signature σ using ${\mathrm{id}}_{A}$ and other public parameters. If the signature verification holds, Bob sets the common shared session key $K_{B,A}=\mathrm{KDF}\left(\left[sk_{{\mathrm{id}}_{B}}\right]L\right)$ and deletes *L*. Otherwise, the protocol terminates here.

The proposed one-pass authenticated key establishment protocol was proved to be secure in the identity-based extended Canetti-Krawczyk (ID-eCK) model [17] in the random oracle model under the ECCDHA [15]. We will use the above algorithm in our proposed handover authentication scheme to establish the common session key between the roaming MN and the target AP.

## 3. Cryptanalysis of He et al. PairHand

He et al.’s PairHand consists of four phases: system initialization phase, handover authentication phase, batch authentication phase, and denial-of-service (DoS) attack resistance phase. In the following, we only briefly review the first two phases of the PairHand, and readers may refer to [6] for details.

### 3.1. Review of He et al. PairHand

**System Initialization**: Given a security parameter κ, the AS first generates an elliptic curve group $G_{1}$ of prime order *q* with a generator *P*, a cyclic multiplicative group $G_{2}$ of same prime order *q*, an admissible bilinear map $\hat{e}:G_{1}\times G_{1}\to G_{2}$. Then, the AS chooses $s\overset{\$}{\leftarrow }Z_{q}^{*}$, computes $P_{\mathrm{pub}}=\left[s\right]P$, and sets the master secret key msk=s. Finally, the AS publishes the public parameters $params=\langle G_{1},G_{2},q,\hat{e},P,P_{\mathrm{pub}},H_{3},H_{4},\mathrm{HMAC}\rangle$.

- For each AP with identity ${\mathrm{id}}_{\mathrm{AP}}\in {\left\{0,1\right\}}^{*}$, the AS computes $Q_{\mathrm{AP}}=H_{3}\left({\mathrm{id}}_{\mathrm{AP}}\right)$ and $d_{\mathrm{AP}}=\left[s\right]Q_{\mathrm{AP}}$. Then, the AS sends the AP’s identity-based private key $d_{\mathrm{AP}}$ to the AP via a secure channel.
- For an MN *i* with identity ${\mathrm{id}}_{i}\in {\left\{0,1\right\}}^{*}$, the AS first checks MN *i*’s validity. If MN *i* is valid, the AS chooses a family of unlinkable pseudo-identities $\mathrm{PID}=\{{\mathrm{pid}}_{1},{\mathrm{pid}}_{2},\ldots ,{\mathrm{pid}}_{\ell }\}$, and for each pseudo-identity ${\mathrm{pid}}_{j}\in \mathrm{PID}$, the AS computes $H_{3}\left({\mathrm{pid}}_{j}\right)$ as the MN *j*’s identity-based public key, and the associated private key $\left[s\right]H_{3}\left({\mathrm{pid}}_{j}\right)$. Then, the AS sends all tuples $\langle H_{3}\left({\mathrm{pid}}_{j}\right),\left[s\right]H_{3}\left({\mathrm{pid}}_{j}\right)\rangle$ back to MN *i* via a secure channel.

**Handover Authentication**: The handover authentication phase is carried out between an MN, say *i*, and an AP, when the AP is within MN *i*’s direct communication range.

1. ${\mathrm{MN}}_{i}\to \mathrm{AP}$: MN *i* picks an unused pseudo-identity ${\mathrm{pid}}_{i}$ and the corresponding private key $\left[s\right]H_{3}\left({\mathrm{pid}}_{i}\right)$. Then MN *i* computes the signature $\sigma_{i}=\left[H_{4}\left(msg_{i}\right)\right]\left[s\right]H_{3}\left({\mathrm{pid}}_{i}\right)$, where $msg_{i}=({\mathrm{pid}}_{i}∥{\mathrm{id}}_{\mathrm{AP}}∥\mathrm{ts})$, a time-stamp ts is added by MN *i* to counter replay attacks, and ‖ indicates message concatenation operation. Subsequently, MN *i* unicasts the access request message $\langle msg_{i},\sigma_{i}\rangle$ to the AP. After that, MN *i* computes the shared session key with the AP as $K_{i}=\hat{e}\left(\left[s\right]H_{3}\left({\mathrm{pid}}_{i}\right),H_{3}\left({\mathrm{id}}_{\mathrm{AP}}\right)\right)$.
2. $\mathrm{AP}\to {\mathrm{MN}}_{i}$: Upon receiving $\langle msg_{i},\sigma_{i}\rangle$, the AP firstly checks whether the time-stamp ts is valid. If ts is invalid, the request will be rejected. Otherwise, the AP verifies the signature $\sigma_{i}$ by checking whether the following equation holds or not:
  $$\hat{e}\left(\sigma_{i},P\right)=\hat{e}\left(\left[H_{4}\left(msg_{i}\right)\right]H_{3}\left({\mathrm{pid}}_{i}\right),P_{\mathrm{pub}}\right).$$
  If it holds, the AP further computes $K_{i}^{\prime }=\hat{e}\left(H_{3}\left({\mathrm{pid}}_{i}\right),\left[s\right]H_{3}\left({\mathrm{id}}_{\mathrm{AP}}\right)\right)$, and generates a message authentication code $tag=\mathrm{HMAC}(K_{i}^{\prime },{\mathrm{pid}}_{i}∥{\mathrm{id}}_{\mathrm{AP}})$. Finally, the AP sends the tuple $\langle {\mathrm{pid}}_{i},{\mathrm{id}}_{\mathrm{AP}},tag\rangle$ to MN *i*.
3. Upon receiving the response $\langle {\mathrm{pid}}_{i},{\mathrm{id}}_{\mathrm{AP}},tag\rangle$ from the AP, MN *i* generates a new message authentication code $tag^{\prime }=\mathrm{HMAC}\left(K_{i},{\mathrm{pid}}_{i}∥{\mathrm{id}}_{\mathrm{AP}}\right)$ and compares it with tag. If $tag^{\prime }$ matches tag, then MN *i* believes the AP is legitimate and has established the shared session key $K_{i}$. Otherwise, MN *i* rejects the connection.
4. AP→AS: Finally, the AP securely transmits $\langle msg_{i},\sigma_{i}\rangle$ to the AS. Upon receiving this message, the AS can find the real identity of MN *i* according to the pseudo-identity included in $msg_{i}$.

### 3.2. Cryptanalysis of He et al. PairHand

He et al. [6] claimed that the signature $\sigma_{i}$ cannot be forged without rigorous security proofs. They soon described a key compromise attack [7] when an adversary obtains a valid signature $\langle msg_{i},\sigma_{i}\rangle$: an adversary can compute $H_{4}{\left(msg_{i}\right)}^{-1}\;\mathrm{mod}\;q$ according to the extended Euclidean algorithm, and can further recover MN *i*’s private key by computing $H_{4}{\left(msg_{i}\right)}^{-1}\sigma_{i}=\left[s\right]H_{3}\left({\mathrm{pid}}_{i}\right)$.

He et al. [7] mistakenly believed that if $H_{4}\left(msg_{i}\right)$ and *q* are not coprime, then an adversary cannot compute the private key $\left[s\right]H_{3}\left({\mathrm{pid}}_{i}\right)$. To remedy the above vulnerability, they suggested the use of composite order bilinear groups instead of prime order bilinear groups, i.e., to fix *q* to be a composite number *n*. Obviously, this will result in lower efficiency because computing the pairing itself becomes significantly slower and also the representation of the group elements becomes substantially longer. More seriously, if $\gcd(H_{4}\left(msg_{i}\right),n)\neq 1$, then *n* is decomposed.

Yeo et al. [8] showed that He et al.’s improved PairHand [7] is still vulnerable to key compromise attack: assume that an adversary gets t>1 messages and their corresponding signatures using the same MN’s private key, i.e., adversary have $\langle msg_{i}^{1},\sigma_{i}^{1}\rangle$, $\langle msg_{i}^{2},\sigma_{i}^{2}\rangle$, …, $\langle msg_{i}^{t},\sigma_{i}^{t}\rangle$. For $1\leq j_{1}<j_{2}\leq t$, thus, the adversary can compute $\gamma =H_{4}\left(msg_{i}^{j_{1}}\right)+H_{4}\left(msg_{i}^{j_{2}}\right)$ and check whether γ is coprime to *n* or not. If adversary finds γ is coprime to *n* for some $1\leq j_{1}<j_{2}\leq t$, then adversary can compute $\gamma^{-1}\left[\sigma_{i}^{j_{1}}+\sigma_{i}^{j_{2}}\right]=\left[s\right]H_{3}\left({\mathrm{pid}}_{i}\right)$. Otherwise, adversary can compute $\gamma =H_{4}\left(msg_{i}^{j_{1}}\right)+H_{4}\left(msg_{i}^{j_{2}}\right)+H_{4}\left(msg_{i}^{j_{3}}\right)$ for all $1\leq j_{1}<j_{2}<j_{3}\leq t$, and check whether γ is coprime to *n* or not. The adversary can repeat the procedure for all sub-combinations of $H_{4}\left(msg_{i}^{1}\right)$, $H_{4}\left(msg_{i}^{2}\right)$, …, $H_{4}\left(msg_{i}^{t}\right)$ until $\left[s\right]H_{3}\left({\mathrm{pid}}_{i}\right)$ is obtained or all combinations are exhausted.

Unfortunately, Yeo et al. [8] did not explain why the improved PairHand is vulnerable to key compromise attack, and give any remedy against it. In fact, if $H_{4}\left(msg_{i}\right)\notin Z_{n}^{*}$ (the probability that a random integer in $Z_{n}$ is not coprime to *n* is equal to φ(n)/(n−1), where φ(n) is the Euler totient function. Obviously, it is not negligible.), then composite number *n* is decomposed. Otherwise, adversary can compute $H_{4}{\left(msg_{i}\right)}^{-1}\;\mathrm{mod}\;n$ from $H_{4}\left(msg_{i}\right)\in Z_{n}^{*}$ in polynomial time by using the extended Euclidean algorithm. Thus, adversary can obtain MN *i*’s identity-based private key $\left[s\right]H_{3}\left({\mathrm{pid}}_{i}\right)$ from $\left[H_{4}\left(msg_{i}\right)\right]\left[s\right]H_{3}\left({\mathrm{pid}}_{i}\right)$ by multiplying $H_{4}{\left(msg_{i}\right)}^{-1}\;\mathrm{mod}\;n$.

The session key established between the AP and MN *i* is $K_{i}=\hat{e}{\left(H_{3}\left({\mathrm{pid}}_{j}\right),H_{3}\left({\mathrm{id}}_{\mathrm{AP}}\right)\right)}^{s}$ in both PairHand and improved PairHand, which is fixed for the same pseudo-identity ${\mathrm{pid}}_{j}$ chosen by MN *i*. This shows that both PairHand and improved PairHand can not achieve forward secrecy. In addition, these pseudo-identities, instead of the MN’s real identity, are used in handover authentication phase for the purpose of privacy protection. Obviously, the AS can link MN *i*’s pseudonyms with its real identity because MN *i*’s pseudonyms are generated by the AS.

## 4. Our Proposed Handover Authentication Scheme

Our proposed handover authentication scheme also consists of four phases: system initialization phase, handover authentication phase, batch authentication phase and DoS attack resistance phase. In order to defend against DoS attack, the method in [6] can be adopted in our scheme. Therefore, we only briefly review the other three phases as follows.

**System Initialization**: Given a security parameter κ, the AS first generates an elliptic curve group $G_{1}$ of prime order *q* with a generator *P*. Then, the AS chooses $s\overset{\$}{\leftarrow }Z_{q}^{*}$ and computes $P_{\mathrm{pub}}=\left[s\right]P$. Finally, the AS sets the master secret key msk=s, and publishes the master public key $mpk=\langle G_{1},q,P,P_{\mathrm{pub}},H_{1},H_{2},H_{3},\mathrm{KDF}\rangle$.

**Figure 2:**
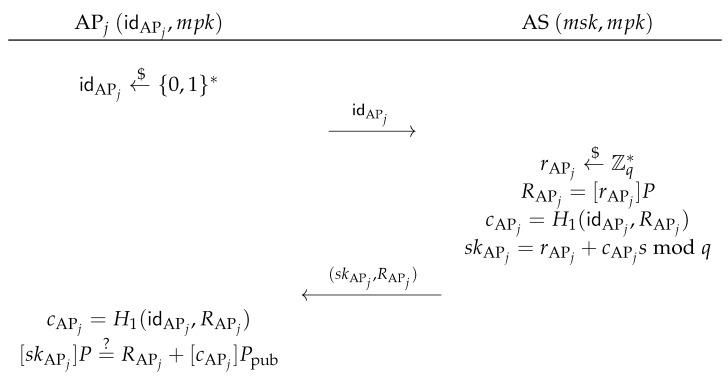
Access point registration phase.

**Figure 3:**
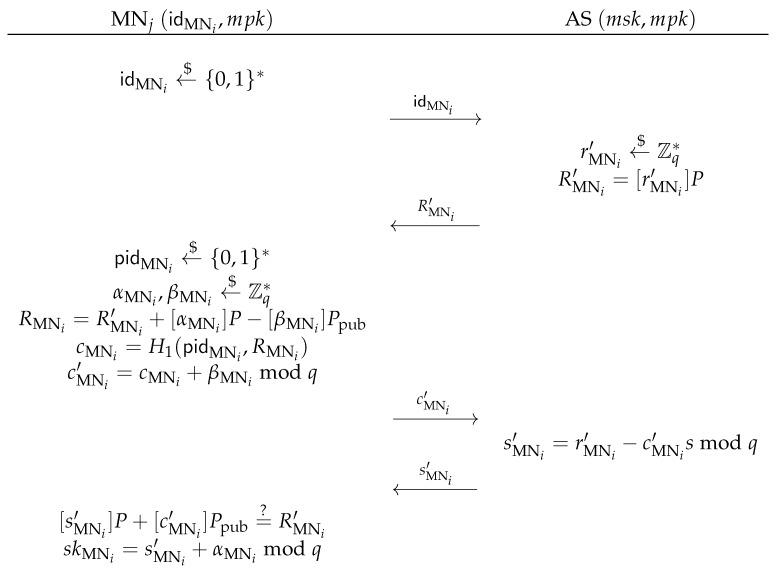
Mobile node registration phase.

- As shown in Figure 2, the AP registration phase is invoked whenever an AP, say *j*, registers to the AS. AP *j* picks an identity ${\mathrm{id}}_{{\mathrm{AP}}_{j}}\overset{\$}{\leftarrow }{\left\{0,1\right\}}^{*}$, and sends ${\mathrm{id}}_{{\mathrm{AP}}_{j}}$ to the AS. Upon receiving the private key request from AP *j*, the AS first chooses $r_{{\mathrm{AP}}_{j}}\overset{\$}{\leftarrow }Z_{q}^{*}$, computes $R_{{\mathrm{AP}}_{j}}=\left[r_{{\mathrm{AP}}_{j}}\right]P$, $c_{{\mathrm{AP}}_{j}}=H_{1}\left({\mathrm{id}}_{{\mathrm{AP}}_{j}},R_{{\mathrm{AP}}_{j}}\right)$, and $sk_{{\mathrm{AP}}_{j}}=r_{{\mathrm{AP}}_{j}}+c_{{\mathrm{AP}}_{j}}s\;\mathrm{mod}\;q$. Then, the AS sends $\left(sk_{{\mathrm{AP}}_{j}},R_{{\mathrm{AP}}_{j}}\right)$ to the AP *j* via a secure channel. Upon receiving the response message from the AS, the AP *j* computes $c_{{\mathrm{AP}}_{j}}=H_{1}\left({\mathrm{id}}_{{\mathrm{AP}}_{j}},R_{{\mathrm{AP}}_{j}}\right)$ and checks the following equation:
  $$\left[sk_{{\mathrm{AP}}_{j}}\right]P\overset{?}{=}R_{{\mathrm{AP}}_{j}}+\left[c_{{\mathrm{AP}}_{j}}\right]P_{\mathrm{pub}}.$$
  If it holds, the AP *j* stores the tuple $\left({\mathrm{id}}_{{\mathrm{AP}}_{j}},sk_{{\mathrm{AP}}_{j}},R_{{\mathrm{AP}}_{j}}\right)$.
- As shown in Figure 3, the MN registration phase is invoked whenever an MN, say *i*, registers to the AS with an identity ${\mathrm{id}}_{{\mathrm{MN}}_{i}}\in {\left\{0,1\right\}}^{*}$, the AS first checks MN *i*’s validity. If MN *i* is valid, the AS chooses $r_{{\mathrm{MN}}_{i}}^{\prime }\overset{\$}{\leftarrow }Z_{q}^{*}$, computes and sends the commitment $R_{{\mathrm{MN}}_{i}}^{\prime }=\left[r_{{\mathrm{MN}}_{i}}^{\prime }\right]P$ to MN *i*. Upon receiving the commitment, MN *i* chooses a pseudonym ${\mathrm{pid}}_{{\mathrm{MN}}_{i}}\overset{\$}{\leftarrow }{\left\{0,1\right\}}^{*}$, blinds $R_{{\mathrm{MN}}_{i}}^{\prime }$ with two random elements $\alpha_{{\mathrm{MN}}_{i}}\overset{\$}{\leftarrow }Z_{q}^{*}$ and $\beta_{{\mathrm{MN}}_{i}}\overset{\$}{\leftarrow }Z_{q}^{*}$, into $R_{{\mathrm{MN}}_{i}}=R_{{\mathrm{MN}}_{i}}^{\prime }+\left[\alpha_{{\mathrm{MN}}_{i}}\right]P-\left[\beta_{{\mathrm{MN}}_{i}}\right]P_{\mathrm{pub}}$, computes $c_{{\mathrm{MN}}_{i}}=H_{1}\left({\mathrm{pid}}_{{\mathrm{MN}}_{i}},R_{{\mathrm{MN}}_{i}}\right)$ and sends the challenge $c_{{\mathrm{MN}}_{i}}^{\prime }=c_{{\mathrm{MN}}_{i}}+\beta_{{\mathrm{MN}}_{i}}\;\mathrm{mod}\;q$ to the AS. Then, the AS returns a values $s_{{\mathrm{MN}}_{i}}^{\prime }=r_{{\mathrm{MN}}_{i}}^{\prime }-c_{{\mathrm{MN}}_{i}}^{\prime }s\;\mathrm{mod}\;q$ to MN *i*. Finally, MN *i* verifies the following equation:
  $$\left[s_{{\mathrm{MN}}_{i}}^{\prime }\right]P+\left[c_{{\mathrm{MN}}_{i}}^{\prime }\right]P_{\mathrm{pub}}\overset{?}{=}R_{{\mathrm{MN}}_{i}}^{\prime }.$$
  If it holds, MN *i* computes $sk_{{\mathrm{MN}}_{i}}=s_{{\mathrm{MN}}_{i}}^{\prime }+\alpha_{{\mathrm{MN}}_{i}}\;\mathrm{mod}\;q$, and obtains MN *i*’s identity-based signing private key $sk_{{\mathrm{MN}}_{i}}$ and public key $R_{{\mathrm{MN}}_{i}}$, which is actually a blind signature that has been signed by the AS for the unknown pseudonym ${\mathrm{pid}}_{{\mathrm{MN}}_{i}}$.
  Notice that the MN *i* can choose a family of unlinkable pseudo-identities ${\mathrm{pid}}_{{\mathrm{MN}}_{i}}^{\left(\ell \right)}\overset{\$}{\leftarrow }{\left\{0,1\right\}}^{*}$, and get the corresponding identity-based signing private keys $sk_{{\mathrm{MN}}_{i}}^{\left(\ell \right)}$ from the AS by choosing $\alpha_{{\mathrm{MN}}_{i}}^{\left(\ell \right)}\overset{\$}{\leftarrow }Z_{q}^{*}$ and $\beta_{{\mathrm{MN}}_{i}}^{\left(\ell \right)}\overset{\$}{\leftarrow }Z_{q}^{*}$, where ℓ=1,2,3,⋯. Thus, the MN *i* can constantly change its pseudo-ID to achieve identity privacy and location privacy in the handover authentication phase.

**Figure 4:**
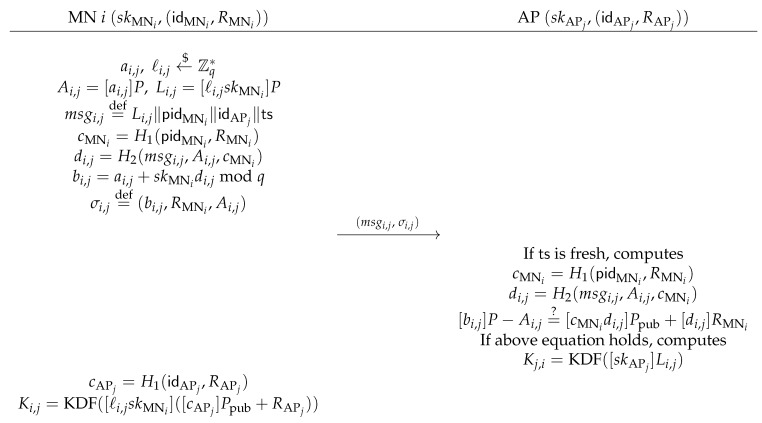
Handover authentication phase.

**Handover Authentication**: When a roaming MN moves out of the coverage of current associated AP, it should handover to a new AP. Assume each AP periodically broadcasts a beacon message, which includes the AP’s certificate together with other necessary network information. The AP’s certificate contains $\left({\mathrm{id}}_{\mathrm{AP}},R_{\mathrm{AP}}\right)$, signed by a trusted certificate authority, and the certificate cannot be impersonated. If a roaming MN *i* chooses a target AP *j*, firstly, MN *i* verifies AP *j*’s certificate to make sure the validity of $\left({\mathrm{id}}_{\mathrm{AP}},R_{\mathrm{AP}}\right)$. Only if validation is successful, MN *i* enters into the handover authentication phase. The detailed description of this phase are as follows, and Figure 4 further illustrates this phase.

1. ${\mathrm{MN}}_{i}\to {\mathrm{AP}}_{j}$: MN *i* with a tuple of pseudo-identity and private key $\left({\mathrm{pid}}_{{\mathrm{MN}}_{i}},sk_{{\mathrm{MN}}_{i}},R_{{\mathrm{MN}}_{i}}\right)$ first chooses $a_{i,j}\overset{\$}{\leftarrow }Z_{q}^{*}$ and $\ell_{i,j}\overset{\$}{\leftarrow }Z_{q}^{*}$, computes $A_{i,j}=\left[a_{i,j}\right]P$, $L_{i,j}=\left[\ell_{i,j}\right]\left[sk_{{\mathrm{MN}}_{i}}\right]P$ and $c_{{\mathrm{MN}}_{i}}=H_{1}\left({\mathrm{pid}}_{{\mathrm{MN}}_{i}},R_{{\mathrm{MN}}_{i}}\right)$. Then, MN *i* sets message $msg_{i,j}=L_{i,j}∥{\mathrm{pid}}_{{\mathrm{MN}}_{i}}∥{\mathrm{id}}_{{\mathrm{AP}}_{j}}∥\mathrm{ts}$, where ts is the time-stamp of the MN *i*. Subsequently, MN *i* computes $d_{i,j}=H_{2}\left(msg_{i,j},A_{i,j},c_{{\mathrm{MN}}_{i}}\right)$, $b_{i,j}=a_{i,j}+sk_{{\mathrm{MN}}_{i}}d_{i,j}\;\mathrm{mod}\;q$, and sets the signature $\sigma_{i,j}=\left(b_{i,j},R_{{\mathrm{MN}}_{i}},A_{i,j}\right)$. At the same time, MN *i* can compute $c_{{\mathrm{MN}}_{i}}=H_{1}\left({\mathrm{id}}_{{\mathrm{AP}}_{j}},R_{{\mathrm{AP}}_{j}}\right)$, and sets the session key $K_{i,j}=\mathrm{KDF}\left(\left[\ell_{i,j}sk_{{\mathrm{MN}}_{i}}\right]\left(\left[c_{{\mathrm{MN}}_{i}}\right]P_{\mathrm{pub}}+R_{{\mathrm{AP}}_{j}}\right)\right)$. Finally, MN *i* sends the handover authentication request $\langle msg_{i,j},\sigma_{i,j}\rangle$ to the target AP *j*.
2. ${\mathrm{AP}}_{j}\to {\mathrm{MN}}_{i}$: Upon receiving the handover authentication request $\langle msg_{i,j},\sigma_{i,j}\rangle$ from an MN *i*, the target AP *j* checks the time-stamp ts. If ts is fresh, the AP computes $c_{{\mathrm{MN}}_{i}}=H_{1}\left({\mathrm{pid}}_{{\mathrm{MN}}_{i}},R_{{\mathrm{MN}}_{i}}\right)$ and $d_{i,j}=H_{2}\left(msg_{i,j},A_{i,j},c_{{\mathrm{MN}}_{i}}\right)$, the AP *j* is able to verify the signature by checking the following equation:
  $$\left[b_{i,j}\right]P-A_{i,j}\overset{?}{=}\left[c_{{\mathrm{MN}}_{i}}d_{i,j}\right]P_{\mathrm{pub}}+\left[d_{i,j}\right]R_{{\mathrm{MN}}_{i}}.$$
  If the above equation does not hold, it implies the message may not sent by a valid MN. Hence, the protocol is terminated at this stage. Otherwise, the AP *j* accepts the message. Finally, the AP *j* computes the symmetric session key $K_{j,i}=\mathrm{KDF}\left(\left[sk_{{\mathrm{AP}}_{j}}\right]L_{i,j}\right)$ using its own private key $sk_{{\mathrm{AP}}_{j}}$.

It is easy to see that if the two parties successfully complete matching sessions, they both compute the same session key:

$$\begin{array}{cc} K_{i,j} & =\mathrm{KDF}\left(\left[\ell_{i,j}sk_{{\mathrm{MN}}_{i}}\right]\left(\left[c_{{\mathrm{AP}}_{j}}\right]P_{\mathrm{pub}}+R_{{\mathrm{AP}}_{j}}\right)\right)=\mathrm{KDF}\left(\left[\ell_{i,j}sk_{{\mathrm{MN}}_{i}}\right]\left[sk_{\mathrm{AP}}\right]P\right)\\ & =\mathrm{KDF}\left(\left[sk_{{\mathrm{AP}}_{j}}\right]\left[\ell_{i,j}sk_{{\mathrm{MN}}_{i}}\right]P\right)=\mathrm{KDF}\left(\left[sk_{{\mathrm{AP}}_{j}}\right]L_{i,j}\right)=K_{j,i}. \end{array}$$

**Batch Verification**: A mass of signature verifications is likely to cause the potential bottleneck at each AP. It is a desirable feature to provide batch verification to solve the problem, which allows an AP to verify multiple signatures simultaneously. Its advantage lies in that the total computation cost in the verification performed by an AP can be apparently reduced.

Our proposed scheme still enjoys the batch verification feature. Assume that an AP *j* receives *n* distinct handover authentication request from *n* distinct MNs, which are denoted as $\langle msg_{1,j},\sigma_{1,j}\rangle$, $\langle msg_{2,j},\sigma_{2,j}\rangle$, …, $\langle msg_{n,j},\sigma_{n,j}\rangle$, respectively. Instead of verifying each individual signature separately, AP *j* can verify these *n* signatures simultaneously by checking the following batch verification criterion:

$$\sum_{i=1}^{n}\left[b_{i,j}\right]P=\sum_{i=1}^{n}\left[c_{{\mathrm{MN}}_{i}}d_{i,j}\right]P_{\mathrm{pub}}+\sum_{i=1}^{n}\left(A_{i,j}+\left[d_{i,j}\right]R_{{\mathrm{MN}}_{i}}\right).$$

It is obvious that, in order to verify these *n* signatures according to the batch verification criterion, AP *j* requires n+2 scalar multiplication over elliptic curve group $G_{1}$. However, if AP *j* verifies each individual signature separately, it requires 3n scalar multiplication over elliptic curve group $G_{1}$.

## 5. Security and Efficiency Analysis

In this section, we give security and efficiency analysis of our proposed handover authentication scheme.

**Theorem** **1.** The proposed handover authentication scheme is ID-eCK secure authenticated key establishment protocol under the ECCDHA in the random oracle model.

**Proof.**  In the handover authentication phase, the roaming MN and the target AP actually perform Yasmin et al.’s one-pass identity-based authenticated key establishment protocol [15], which is proved to be ID-eCK secure under the elliptic curve computational Diffie–Hellman assumption in the random oracle model. ☐

In the following, we provide an informal discussion on security properties that are satisfied by our proposed handover authentication scheme.

- **MN’s Anonymity and Untraceability**: In existing handover authentication schemes using identity-based signature schemes, to guarantee MN’s privacy, the AS chooses a family of pseudo-identities and generates associated private keys for each MN. Undoubtedly, the AS knows the relationship between each MN’s pseudonyms and real identity. More seriously, the AS knows MN’s private keys, this is known as the key escrow problem in identity-based cryptography. In our proposed scheme, each MN can choose a family of pseudonyms and and obtain associated private keys by running Pointcheval and Stern’s blind signature scheme with the AS in the registration phase. Although the handover authentication request messages must include a pseudonym of the roaming MN; however, there is no linkage between these pseudonyms, anyone, even the AS, does not know the MN’s private keys, is unable to identify the MN or to link two sessions initiated by the same MN (i.e., trace the movement routes of the MN). Thus, our proposed handover authentication scheme is escrow-free and achieves MN’s anonymity and untraceability.
- **MN’s Key Compromise Security**: In the handover authentication phase, the access request sent by MN *i* to AP *j* is actually a signature that generated by MN *i* with its signing private key on the message $msg_{i,j}={\mathrm{pid}}_{{\mathrm{MN}}_{i}}∥{\mathrm{id}}_{{\mathrm{AP}}_{j}}∥\mathrm{ts}$, which is used to prove to AP *j* that MN *i* is the private key holer corresponding to the pseudonym ${\mathrm{pid}}_{{\mathrm{MN}}_{i}}$. Here, we use the GG-IBS scheme. One reason for this is its efficiency and simplicity, and another more important reason is that it has been proved to be EUF-ID-CMA secure in the random oracle model under the ECDLA. Even if an adversary gets t>1 messages and their corresponding signatures generated by the same MN *i*, he can not forge a valid signature of MN *i*, let alone get MN *i*’s private key. Thus, our proposed scheme can resist MN’s key compromise attack.
- **MN’s Forward Secrecy**: The session key $K_{i,j}=\mathrm{KDF}\left(\left[\ell_{i,j}sk_{{\mathrm{MN}}_{i}}\right]\left(\left[c_{{\mathrm{AP}}_{j}}\right]P_{\mathrm{pub}}+R_{{\mathrm{AP}}_{j}}\right)\right)$ calculated by MN *i* is equal to the session key $K_{j,i}=\mathrm{KDF}\left(\left[sk_{{\mathrm{AP}}_{j}}\right]L_{i,j}\right)$ calculated by Ap *j*. According to the ECCDHA, there is no probabilistic polynomial-time adversary can compute the session key without MN *i*’s private key or AP *j*’s private key. Unlike PairHand, where the session key is fixed, the session key in our proposed scheme is random that depends on two random elements $a_{i,j}\overset{\$}{\leftarrow }Z_{q}^{*}$ and $\ell_{i,j}\overset{\$}{\leftarrow }Z_{q}^{*}$ chosen by MN *i*. Thus, our proposed scheme achieves MN’s forward secrecy.

Next, we compared our proposed handover authentication scheme with other existing handover authentication schemes [7,9,10,11,12] in terms of security, communication round, computation cost and bandwidth requirement. The results of this comparison are shown in Table 2 below.

For security, our proposed scheme is key escrow-free and achieves anonymity and untraceability for MNs, while schemes in [7,9,10,11,12] have an inherent drawback of key escrow problem, and can only provide weak anonymity and untraceability for MNs. He et al.’s scheme [7] is vulnerable to key compromise attack for MNs, while schemes in [9,10,11,12] and ours can resist the attack. Schemes in [11,12] and ours enjoy forward secrecy for MNs, while schemes in [7,9,10] do not.

Reducing communication cost is extremely important in wireless networks, Barr and Asanovi [18] pointed out wireless transmission of a bit can require over 1000 times more energy than a single 32-bit computation. To establish a shared session key between MN and AP, there are two message transmissions in existing handover authentication schemes [7,9,10,11,12], while there is only one message transmission in our proposed scheme.

For computational cost, we focus on the time spent on the high cost operations, such as the time ($T_{\mathrm{bp}}$) spent on the bilinear pairing operations over $G_{1}\times G_{1}$, the time ($T_{\mathrm{sm}}$) spent on the scalar multiplications over the elliptic curve group $G_{1}$, while the time spent on highly efficient operations, such as the hash function and key derivation function, is neglected. Both MN and AP need to perform complicated bilinear pairings in [7,9,10], while there is no bilinear pairing operation in [11,12] and our proposed scheme. Moreover, both Li et al.’s scheme [11] and Chaudhry et al.’s scheme [12] do not enjoy batch verification function, but our proposed scheme does.

To evaluate bandwidth requirement, we assume that the size of a time-stamp, the length of the pseudo identity of MNs and the identity of APs are 32 bits, 128 bits, and 128 bits, respectively. It is well known that 3072-bit RSA keys are equivalent in strength to 128-bit symmetric keys and 256-bit elliptic curve cryptography keys. To provide 128-bit security, one can choose 256-bit prime order elliptic curve group $G_{1}$ in [9,10,11,12] and our proposed scheme, while one needs to choose 3072-bit prime order elliptic curve group $G_{1}$ in [7]. In [7], the authentication request packet consists of MN’s pseudo identity, AP’s identity, time-stamp and one element in $G_{1}$, and the authentication response packet consists of MN’s pseudo identity, AP’s identity, and one element in $Z_{q}^{*}$. The total communication cost of He et al.’s scheme is 3872 bits. In [9], the authentication request packet consists of MN’s pseudo identity, AP’s identity, time-stamp and two elements in $G_{1}$, and the authentication response packet consists of MN’s pseudo identity, AP’s identity, one element in $Z_{q}^{*}$. The total communication cost of Tsai et al.’s scheme is 1312 bits. In [10], the authentication request packet consists of MN’s pseudo identity, AP’s identity, time-stamp and two elements in $G_{1}$, and the authentication response packet consists of MN’s pseudo identity, AP’s identity, and one element in $Z_{q}^{*}$. The total communication cost of Wang et al.’s scheme is 1312 bits. In [11], the authentication request packet consists of MN’s pseudo identity, AP’s identity, time-stamp, three elements in $G_{1}$ and one element in $Z_{q}^{*}$, and the authentication response packet consists of MN’s pseudo identity, AP’s identity, one element in $G_{1}$ and one element in $Z_{q}^{*}$. The total communication cost of Li et al.’s scheme is 2080 bits. In [12], the authentication request packet consists of MN’s pseudo identity, AP’s identity, time-stamp, two elements in $G_{1}$ and one element in $Z_{q}^{*}$, and the authentication response packet consists of MN’s pseudo identity, AP’s identity, one element in $G_{1}$ and one element in $Z_{q}^{*}$. The total communication cost of Chaudhry et al.’s scheme is 1824 bits. In our proposed scheme, the authentication request packet consists of MN’s pseudo identity, AP’s identity, time-stamp, three elements in $G_{1}$ and one element in $Z_{q}^{*}$, and the total communication cost of our proposed scheme is 1312 bits.

In summary, our proposed scheme has advantages in security, communication and computation in comparison with existing handover authentication schemes [7,9,10,11,12].

## 6. Conclusions

A fast handover authentication scheme is essential to seamless services for delay sensitive applications in wireless networks. At the same time, data security and user privacy have become increasingly important in mobile computing, particularly in the context of handover authentication schemes as they relate to users’ credential information. In this paper, we first show that He et al.’s handover authentication scheme does not meet the main security properties: key compromise security, forward secrecy, escrow-free and strong anonymity for mobile nodes. Then, we propose a new secure and efficient handover authentication scheme using elliptic curve cryptography. Not only does the proposed scheme satisfy all the essential security requirements for handover authentication schemes, but it also achieves forward secrecy, escrow-free and strong anonymity for mobile nodes. The proposed scheme is provably secure under the elliptic curve computational Diffie–Hellman assumption in the random oracle model and outperforms previously reported schemes in terms of computation and communication overhead. There is only one message transmission between a roaming mobile node and the target access point in our proposed scheme, while there are at least two message transmissions between a roaming mobile node and the target access point in other existing schemes. To achieve better performance, it is a desirable feature to provide batch verification where the target access point can verify the correctness of multiple received messages simultaneously. Unfortunately, all previous handover authentication schemes either support batch verification but require complicated bilinear pairing operations, or do not support batch verification but do not require bilinear pairing operations. There is no complicated bilinear pairing operation, and batch verification is also supported in our proposed scheme. Therefore, our proposed scheme is well suited for implementing secure communication in wireless networks. Thus far, all of the existing handover authentication schemes are proved to be secure in the random oracle model. However, Canetti et al. showed that some cryptographic schemes that are provably secure in the random oracle model are completely insecure when the random oracle is instantiated with any function family. It is interesting to design new efficient handover authentication schemes that are provably secure in the standard model.

## Figures and Tables

**Table 1:** The notations used in the proposed scheme.

Symbol	Description
κ	Security parameter
$x\overset{\$}{\leftarrow }S$	Pick an element *x* uniformly at random from the set S
$H_{1}$	A cryptographic secure hash function $H_{1}:{\left\{0,1\right\}}^{*}\times G_{1}\to Z_{q}$
$H_{2}$	A cryptographic secure hash function $H_{2}:{\left\{0,1\right\}}^{*}\times G_{1}\times Z_{q}\to Z_{q}$
$H_{3}$	A cryptographic secure hash function $H_{3}:{\left\{0,1\right\}}^{*}\to G_{1}$
$H_{4}$	A cryptographic secure hash function $H_{4}:{\left\{0,1\right\}}^{*}\to Z_{q}$
HMAC	A cryptographic secure message authentication code function ${\mathrm{HMAC}}_{1}:G_{2}\times {\left\{0,1\right\}}^{*}\to Z_{q}$
KDF	A cryptographic secure session key derivation function $\mathrm{KDF}:G_{1}\to {\left\{0,1\right\}}^{\kappa }$

**Table 2:** Comparison of handover authentication protocols.

	[7]	[9]	[10]	[11]	[12]	**Ours**
MN Computational Cost	$1T_{\mathrm{bp}}+T_{\mathrm{sm}}$	$1T_{\mathrm{bp}}+T_{\mathrm{sm}}$	$1T_{\mathrm{bp}}+T_{\mathrm{sm}}$	$3T_{\mathrm{sm}}$	$3T_{\mathrm{sm}}$	$3T_{\mathrm{sm}}$
AP Computational Cost	$3T_{\mathrm{bp}}+T_{\mathrm{sm}}$	$3T_{\mathrm{bp}}+T_{\mathrm{sm}}$	$3T_{\mathrm{bp}}+T_{\mathrm{sm}}$	$4T_{\mathrm{sm}}$	$6T_{\mathrm{sm}}$	$3T_{\mathrm{sm}}$
Communication Round	2	2	2	2	2	1
Communication Cost	3872 bits	1312 bits	1312 bits	2080 bits	1824 bits	1312 bits
Batch Verification	Yes	Yes	Yes	No	No	Yes
Group Order	Composite	Prime	Prime	Prime	Prime	Prime
MN Anonymity & Untraceability	Weak	Weak	Weak	Weak	Weak	Strong
MN Key Compromise Security	No	Yes	Yes	Yes	Yes	Yes
MN Forward Secrecy	No	No	No	Yes	Yes	Yes
